# METTL3 drives NSCLC metastasis by enhancing CYP19A1 translation and oestrogen synthesis

**DOI:** 10.1186/s13578-024-01194-9

**Published:** 2024-01-18

**Authors:** Wangyang Meng, Han Xiao, Rong Zhao, Jiaping Chen, Yangwei Wang, Peiyuan Mei, Hecheng Li, Yongde Liao

**Affiliations:** 1 Department of Thoracic Surgery, Ruijin Hospital, Shanghai Jiao Tong University School of Medicine, Shanghai, 200025 China; 2 Department of Thoracic Surgery, Union Hospital, Tongji Medical College, Huazhong University of Science and Technology, Wuhan, 430022 China; 3 Department of Thoracic Surgery, Shandong Provincial Hospital Affiliated to Shandong First Medical University, Jinan, 250021 China; 4 Department of Cardiothoracic Surgery, Third Affiliated Hospital of Kunming Medical University (Yunnan Cancer Hospital), Kunming, Yunnan China

**Keywords:** NSCLC, Small molecule inhibitor, METTL3, Translation regulation, Estrogen

## Abstract

**Background:**

METTL3 plays a significant role as a catalytic enzyme in mediating N6-methyladenosine (m^6^A) modification, and its importance in tumour progression has been extensively studied in recent years. However, the precise involvement of METTL3 in the regulation of translation in non-small cell lung cancer (NSCLC) remains unclear.

**Results:**

Here we discovered by clinical investigation that METTL3 expression is correlated with NSCLC metastasis. Ablation of METTL3 in NSCLC cells inhibits invasion and metastasis in vitro and in vivo. Subsequently, through translatomics data mining and experimental validation, we demonstrated that METTL3 enhances the translation of aromatase (CYP19A1), a key enzyme in oestrogen synthesis, thereby promoting oestrogen production and mediating the invasion and metastasis of NSCLC. Mechanistically, METTL3 interacts with translation initiation factors and binds to CYP19A1 mRNA, thus enhancing the translation efficiency of CYP19A1 in an m^6^A-dependent manner. Pharmacological inhibition of METTL3 enzymatic activity or translation initiation factor eIF4E abolishes CYP19A1 protein synthesis.

**Conclusions:**

Our findings indicate the crucial role of METTL3-mediated translation regulation in NSCLC and reveal the significance of METTL3/eIF4E/CYP19A1 signaling as a promising therapeutic target for anti-metastatic strategies against NSCLC.

**Graphical abstract:**

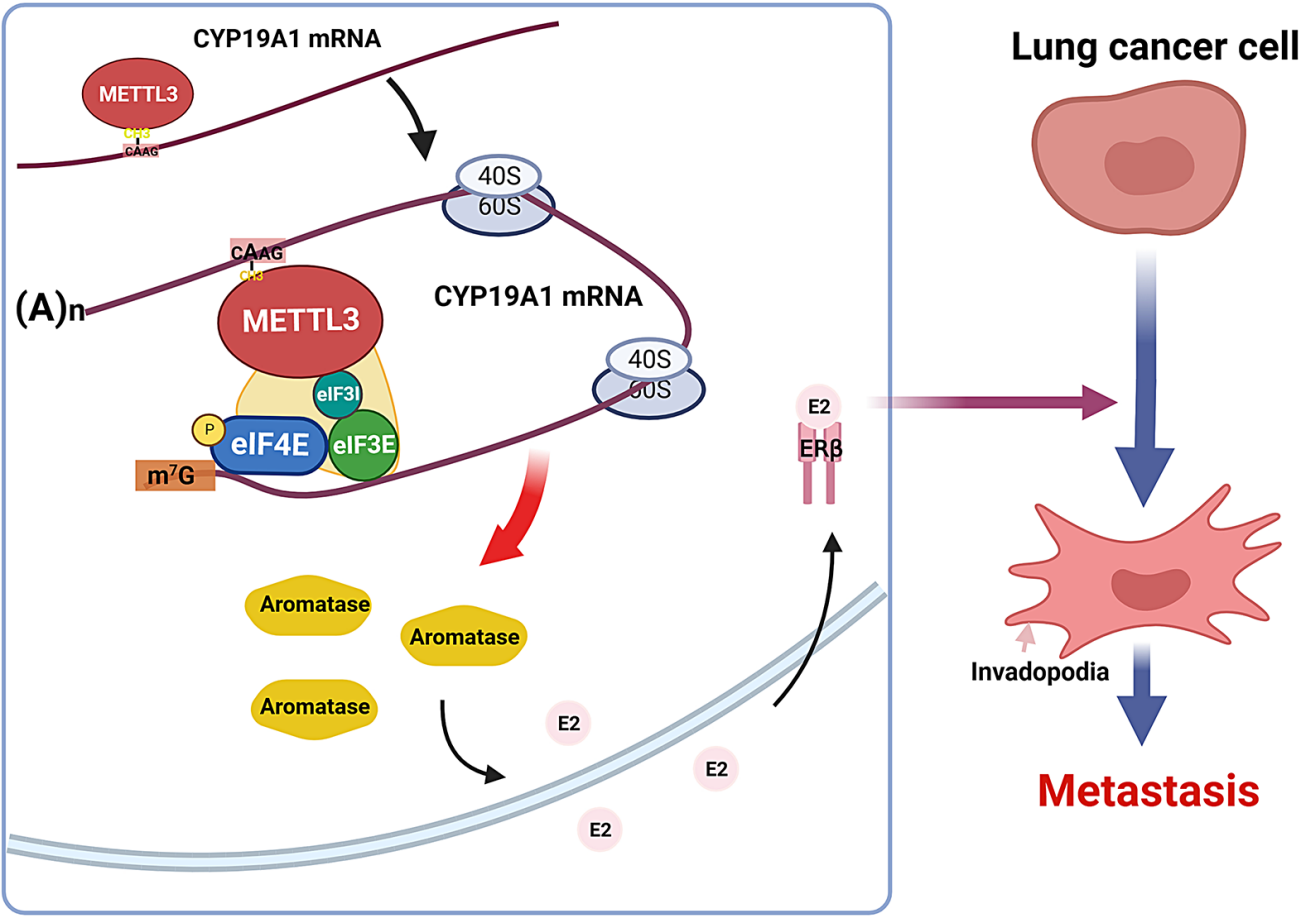

**Supplementary Information:**

The online version contains supplementary material available at 10.1186/s13578-024-01194-9.

## Introduction

Tumour metastasis continues to be a significant concern for a large number of lung cancer patients, posing a risk to their long-term survival [1]. The process of tumour metastasis is complex and the molecular mechanisms underlying the development of lung cancer metastasis remain incompletely understood. Studies have indicated that invadopodia play a crucial role in the extravasation of cancer cells [2, 3]. These specialized structures enable tumour cells to penetrate the extracellular matrix by extending filamentous pseudopod-like projections facilitated by membrane-associated protein hydrolases [4, 5]. Subsequently, these invasive cells enter the circulatory system, forming metastatic foci through colonization [6].

N6-methyladenosine (m^6^A) is the most prevalent chemical modification found in eukaryotic RNA, and it plays crucial regulatory roles in RNA stability, splicing, and translation [7, 8]. METTL3, the primary m^6^A methyltransferase, has garnered significant attention in recent years due to its involvement in various tumours and its association with tumour metastasis [9, 10]. However, the precise molecular mechanism through which METTL3 promotes metastasis in lung cancer remains incompletely understood, and further investigation is warranted to explore the downstream molecular pathways it regulates.

Gregory et al. in Nature in 2018 [11] reported that a significant number of mRNAs exhibit notable changes in translation efficiency following METTL3 depletion. METTL3, when binding to specific mRNA sites near the stop codon, facilitates ribosomal recycling and enhances translation by directly interacting with eukaryotic initiation factor 3 subunit h (eIF3h). Recent findings have indicated that METTL3 primarily enhances translation of epigenetic factors, thereby promoting tumourigenesis [12]. Furthermore, newly reported studies have revealed that METTL16, which belongs to the same family as METTL3, significantly boosts protein translation efficiency [13]. METTL16 interacts with the translation initiation factor eI3a/b, resulting in the enhanced translation efficiency of mRNA.

Our research group has conducted a comprehensive and long-term investigation into the role and underlying mechanism of β-estradiol (E2, one of the three primary oestrogens) and oestrogen receptor beta (ERβ) in the development of non-small cell lung cancer (NSCLC) [14–16]. We have confirmed that ERβ serves as the primary functional receptor for E2, driving metastasis in NSCLC. The activation of the prometastatic effect by the E2/ERβ axis is mainly mediated by ICAM1 and matrix metalloproteinase 2 (MMP2) [17, 18]. Aromatase (CYP19A1), a rate-limiting enzyme involved in oestrogen biosynthesis, plays a crucial role in activating the E2/ERβ signal and also contributes significantly to lung cancer metastasis [19]. Combining CYP19A1 inhibitors with NSAIDs has demonstrated enhanced antitumour effects by reducing circulating E2 levels, proinflammatory cytokines, and macrophage recruitment in the lung microenvironment [20].

In this study, we conducted a systematic evaluation of METTL3 expression in non-small cell lung cancer (NSCLC). Subsequently, we explored the impact of METTL3 on the invasive and migratory abilities of lung cancer cells, along with their invadopodia formation and extracellular matrix degradation capabilities, which are fundamental to their metastatic potential. Furthermore, to assess the effect of METTL3 on translation, we initially analyzed a previously published dataset and identified CYP19A1 as a target of METTL3-mediated translation regulation. Since CYP19A1 plays a critical role in activating E2/ERβ signalling and promoting NSCLC metastasis, we performed in vitro experiments to validate that the expression of CYP19A1, as well as its associated migration and invasion, is dependent on the presence of METTL3. Mechanistically, we discovered that METTL3 interacts with translation initiation factors (eIF4E, eIF3e, eIF3i) and binds to CYP19A1 mRNA, thereby enhancing the translation efficiency of CYP19A1 in an m^6^A-dependent manner. Inhibition of eIF4E impairs the METTL3-mediated enhancement of CYP19A1 translation. Additionally, the use of STM2457, a small-molecule inhibitor of METTL3 enzymatic activity, demonstrated a similar effect to METTL3 knockout regarding CYP19A1 translation. These findings highlight the role of METTL3 in mediating NSCLC metastasis through the augmentation of CYP19A1 translation. Therefore, this study elucidates a novel mechanism by which METTL3 promotes metastasis and provides a new therapeutic strategy for treating NSCLC metastasis.

## Methods

### Patients and NSCLC samples

The clinical specimens utilized in this study were obtained from the Department of Thoracic Surgery at Union Medical College, Huazhong University of Science and Technology in China. The specimens included primary tumours as well as adjacent normal tissue, which were collected from each patient at a distance of more than 2 cm from the edge of the primary tumour. In cases where lymph node metastasis was present, the patient’s metastatic lymph node was also collected. The patients’ clinical data and pathological information were comprehensively gathered and recorded, and their health status was evaluated through regular follow-up visits. All specimens were collected in the operating room and divided into two parts. One part was transferred to paraformaldehyde and stored in specimen collection bags at room temperature, while the other part was immediately transferred to 2 mL freeze storage tubes and stored in an ultra-low temperature − 80 degrees refrigerator for long-term preservation. It is important to note that all specimens used in this study were obtained with the approval of the Medical Ethics Committee of Union Hospital, Tongji Medical College, Huazhong University of Science and Technology (IRB ID number is 20,180,403).

### Cell lines and transfection

The human NSCLC cell lines A549 and H1975 were acquired from the American Type Culture Collection. All cells were cultured in DMEM high glucose medium supplemented with 10% fetal bovine serum (FBS) and antibiotics (100 units/mL penicillin and 100 µg/mL streptomycin, Corning, Cat. No. 30-002-CI). Cell culture flasks and well plates were placed in a cell culture incubator set at 37°C with 5% CO2, and cells were observed daily using an inverted microscope. The small interfering RNA for METTL3 (5’-CTGCAAGTATGTTCACTATGA-3′) was obtained from Riobio in Guangzhou, China. Plasmids for METTL3 overexpression were acquired from Genechem in Shanghai, China. Lipofectamine 3000 (Thermo-Fisher Scientific, Waltham, MA, USA) was used for plasmid and siRNA transfections. For each well in the 6-well dish, 1.5 µg of plasmid DNA and a final siRNA concentration of 40 nM were utilized.

### CRISPR deletion

To perform METTL3 knockdown (KO) in human cells through CRISPR deletion, the A549 cell line was selected. Plasmids carrying the CRISPR/Cas9 system, obtained from Genomeditech in Shanghai, China, were utilized. These plasmids contained a validated sgRNA sequence (sequence: CTGGGCTTAGGGCCACCAG) targeting the METTL3 gene, as previously confirmed by Vu et al. [21]. For plasmid transfection, Lipofectamine® 3000 reagent from Invitrogen in the USA was employed following the manufacturer’s instructions. Subsequently, cells incorporating the CRISPR/Cas9 system were selected using puromycin. The selected cells were then expanded, and total protein extraction was performed at the beginning, midway through the experiments, and before the in vivo assay to confirm downregulation of the METTL3 protein. Phenotypic assays were conducted accordingly. Wild-type cells served as controls, along with cells transfected with a scrambled (non-specific guide) vector, which were used to control for any potential off-target effects in all experiments.

### Gelatin invadopodia assay

The invadopodia test was performed according to the manufacturer’s guidelines (QCMTM Gelatin Residency Test, Millipore). Briefly, fluorescent gelatin is applied to a glass chamber slide already coated with Poly L Lysine and then activated with a dilute glutaraldehyde solution. The slides were then used as a substrate for the endophytic bacteria. The surface of the slides was washed with 70% alcohol and quenched with growth medium without aldehyde, and then the cells were seeded onto the gelatin surface and allowed to grow for 16 h. Afterwards, cells were fixed by adding DPBS containing 4% formaldehyde, and then stained for F-Actin (TRITC-Phalloidin) followed by observation under a fluorescence microscope. The activity of the invadopodia was demonstrated by the clearance of fluorescent matrix proteins from the region of the invadopodia.

### Wound healing assay

The cell density was adjusted to 7*10^5^/mL. The culture insert (Ibidi, Germany) was placed in a 6-well plate and 70 µL of cell suspension was gently added dropwise to the two compartments of the insert. The cells were then placed in a cell culture incubator and incubated overnight. If the cells are stably regulated, they are digested and resuspended for counting at the exponential growth of the cells, and the subsequent operation is the same as the above steps. After overnight incubation, the cells were observed under a light microscope. When the cells have grown to the bottom of the insert, flush out the insert with sterile forceps on an ultra-clean table and gently rinse the 6-well plate twice with PBS. Subsequently, aspirate the PBS and add 2mL of serum-free medium. The moment of adding serum-free medium was recorded as 0 h, and at this moment, observation was performed and images were collected using an inverted light microscope, followed by incubation in an incubator. Observations were made and images were collected at 12 and 24 h. Each group of experiments was repeated three times, and finally the difference of cell migration ability between different groups was judged according to the distance of cell migration.

### Transwell invasion assay

Matrigel (BD, Franklin Lakes, NJ, USA) was diluted to 10x volume with serum-free medium and 30uL was added onto the transwell membrane (Corning, NY, US). After the membrane is gelled, it was placed in the cell culture oven overnight. Then the excess liquid from the chambers was carefully aspirated. The cells were adjusted to 4*10^5^/mL using serum-free medium. 200 µL was aspirated into the chambers and gently puffed into the upper chamber coated with Matrigel. 600µL of medium with 20% serum was added in the lower chamber of 24-well plate. The cells were incubated for 24 ~ 48 h. The chambers were removed and the excess liquid was aspirated from the upper and lower chambers. 600 µL of PBS was added to the 24-well plate, and the chambers were placed in PBS and then fixed in 4% paraformaldehyde for 15 min. After the chambers were dried completely. The cells were gently wiped off the surface of the upper chamber with a cotton swab moistened with PBS. For each sample, three random fields of view were selected and the number of penetrated cells was counted. Each set of experiments was repeated three times and statistically analyzed.

### Scanning electron microscopy (SEM)

Wuhan Institute of Virology, Chinese Academy of Sciences provided support for scanning electron microscopy. Briefly, an electron microscope fixative (Cat. No. G1102–100ML, 113 ServiceBio, Wuhan, China) was employed to fix the METTL3 knockout/overexpressing cells and control cells. The samples were then treated to reach the necessary drying point, followed by mounting, iridium coating, and image capturing.

### Animal experiments

Female BALB/c Nude mice (8 weeks old, two groups) were obtained from Beijing Vital River Laboratory Animal Technology Co. Ltd. The effect of METTL3 on in vivo metastasis was examined by injecting KO-METTL3 or KO-NC A549 cells (4 × 10^6^) into the tail vein on day 0. Mice in each group were sacrificed on day 42, and their lungs and livers were collected for further analysis. Another group of mice, injected with KO-NC and KO-METTL3 cells for the tail-vein injection metastasis model, were kept in a specialized-pathogen-free environment for survival analysis.

### ELISA

A549 cells were digested and resuspended for counting, and seeded in 6-well plates with 1*10^5^ cells per well. The cell culture supernatant was collected and filtered through 0.45 μm filter to exclude cell debris after 3 days and stored in a 4℃ refrigerator for extracellular E2 determination. Then cells were digested and centrifuged and resuspended using complete medium for counting. Subsequently, 120 µL of cell suspension was aspirated into a 1.5 mL EP tube. Cells were fragmented using freeze-thaw fragmentation, a non-mechanical cell fragmentation method in which cells are placed in liquid nitrogen for rapid freezing and subsequently thawed at room temperature. After cell crushing, the tubes were centrifuged at 12,000 rpm for 10 min, and 100 µL of supernatant was aspirated and stored in a 4 °C refrigerator for intracellular E2 determination.

For metastatic lung tumours, tumour interstitial fluid (TIF) was collected as previously described [22]. The concentration of E2 were measured using a Human E2 ELISA Kit (Cusabio, Wuhan, China). All experiments were performed according to the manufacturer’s instructions.

### Immunofluorescence staining

The medium in the confocal dish was discarded and rinsed three times with PBS on a horizontal shaker for 5 min. 500 µL of 4% paraformaldehyde solution was added and subsequently fixed for 30 min at room temperature. After fixation, the dishes were rinsed three times with PBS for 5 min each. The confocal dishes were closed at room temperature for 45 min by adding 1 mL of 5% BSA solution, and then rinsed twice with PBS for 3 min each. 200 µL of TRITC-Phalloidin (1:250, Sigma, Cat. No. P1951) diluted by PBS solution containing 0.1% Triton ×100 and 1% BSA was added to the confocal dish and stained for 1 h. 70 µL of DAPI staining reagent was added and incubated for 10 min at room temperature. Anti-fluorescence quencher was added into the dish. The samples were then stored in a wet box at 4 °C away from light and observed as soon as possible. Images were captured using a Nikon A1 Confocal microscope (Nikon, Tokyo, Japan).

### Immunohistochemistry and scoring

Formalin-fixed, paraffin-embedded tissue was cut into 4 μm sections and analyzed by IHC, as described previously [14]. Briefly, tissue sections were incubated with anti-METTL3 (#ab195352, dil. 1:500, Abcam), anti-Aromatase (#ab18995, dil. 1: 500, Abcam), anti-ESR2 (#14007-1-AP, dil. 1:200, Proteintech) as primary antibodies after heat-induced epitope retrieval, followed by secondary antibody (diluted 1:100, G1213, Servicebio) incubation and diaminobenzidine. Slides were counterstained with hematoxylin. Then the researchers scored the expression levels of METTL3, CYP19A1, and ERβ in a double-blind manner, and the scoring system was rationalized according to the previous description [15].

### Western blot analysis, co-immunoprecipitation (Co-IP)

Tissue and whole cell lysates are extracted by homogenizing tissues or cell pellets in RIPA buffer. For co-immunoprecipitation, lysates containing 500 µg total protein were incubated with specific antibodies (4 µg) at 4℃ for 16 h in continuous rotation. After that, 50 µl protein G agarose beads were added and incubated for 3 h. The precipitated protein was re-suspended in 30 µl SDS sample buffer and boiled at 95 ° C for 10 min. Then the protein was isolated and transferred to the PVDF membrane. The membrane was blocked with 5% bovine serum albumin (BSA, Servicebio, Wuhan) at room temperature for 1 h and labeled with primary antibodies (anti-METTL3, anti-eIF4E, anti-eIF3e, anti-eIF3i) at 4℃ overnight. The membrane was then incubated with a second antibody bound to horseradish peroxidase, and the protein bands were detected with ECL reagents.

### Mass spectrometry and analysis

The retrieved proteins eluted from immunoprecipitation was sent for qualitative protein profiling (BGI Genomics, Shenzhen) to identify the proteins bound to METTL3. Protein samples from the IP group and IgG group were sent separately, with two replicates of each sample. The identified differential proteins were analyzed by GO (Gene Ontology) to classify them into three components: cellular component (CC), molecular function (MF) and biological process (BP), in order to identify the basic functions of the bound proteins. The basic functions of the binding proteins were identified.

### RNA extraction and RT-qPCR

The cell and tissue total RNA was extracted using FastPure Cell/Tissue Total RNA Isolation Kit (Vazyme, Nanjing, China). NanoDrop 2000 spectrophotometer (NanoDrop Technologies, Wilmington, DE, USA) was used for RNA concentration detection. 1000 ng of the RNA was reverse transcribed into cDNA using ABScript III Reverse Transcriptase (ABclonal, Wuhan, China) according to the manufacturer’s instruction. Then RT-qPCR experiment was performed using ChamQ SYBR qPCR Master Mix (Vazyme, Nanjing, China). The primers for qPCR were obtained from TSINGKE (Guangzhou, China), and the primer sequences were as follows (from 5’ to 3’): METTL3: TTGTCTCCAACCTTCCGTAGT (forward) and CCAGATCAGAGAGGTGGTGTAG (reverse); CYP19A1: TGGAAATGCTGAACCCGATAC (forward) and AATTCCCATGCAGTAGCCAGG (reverse); ESR2: AGCACGGCTCCATATACATACC (forward) and TGGACCACTAAAGGAGAAAGGT (reverse); GAPDH: GGAGCGAGATCCCTCCAAAAT (forward) and GGCTGTTGTCATACTTCTCATGG (reverse).

### MeRIP‑qPCR assay

The m^6^A immunoprecipitation (MeRIP) procedure was conducted following the instructions provided by the manufacturer, utilizing a Magna MeRIP™ m^6^A kit (#17-10499, Merck Millipore, MA). In brief, purified mRNA underwent DNase I digestion and was subsequently fragmented into approximately 100nt fragments using an RNA fragmentation reagent, with an incubation step at 94 °C. Following fragmentation, the stop buffer was introduced, and standard ethanol precipitation was performed to collect the fragmented mRNA. To initiate the immunoprecipitation process, the anti-m^6^A antibody (12 µg) was pre-incubated with 50 µl beads in IP buffer (containing 150mM NaCl, 0.1% NP-40, 10mM Tris-HCl at pH 7.4) for 1 h at room temperature. Next, fragment mRNAs were added to the antibody-beads mixture and incubated on a rotator at 4 °C for 4 h. After proper washing steps, the immunoprecipitated mixture was subjected to digestion using a high concentration of proteinase K. The bound RNAs were then extracted using the phenol-chloroform method followed by ethanol precipitation. These extracted RNAs were utilized for subsequent qPCR analysis or library construction. The evaluation of m^6^A modification in CYP19A1 mRNA was performed via qPCR analysis, employing specific primers designed to target the precise regions. Prediction of m^6^A sites within CYP19A1 was accomplished using SRAMP (http://www.cuilab.cn/sramp) [23]. Primers were designed to make sure that the target sequence included m^6^A site within 100nt length as follows:

CYP19A1-Peak-Forward: 5’- GCACACCTTGGAATTCGCTT − 3’;

CYP19A1-Peak-Reverse: 5’- GTGGTGTTTGGAAAGTTCCTCC − 3’.

### RNA immunoprecipitation (RIP) assay

RIP assays were performed using an Imprint® RNA Immunoprecipitation (RIP) Kit (Sigma-Aldrich, USA) according to the manufacturer’s instructions. Cells at approximately 90% confluence was lysed using complete RIP lysis buffer containing RNase Inhibitor and protease inhibitor and then 100 µl of whole cell extract was incubated with RIP buffer containing magnetic beads conjugated to specific antibodies. The negative control was normal mouse anti-IgG antibody (#2729, CST), and the positive control was anti-METTL3 antibody (#ab195352, Abcam).

### Antibodies and reagents

Primary antibodies for western blot or IHC include anti-METTL3 (#ab195352, Abcam), anti-GAPDH (#60,004–1-Ig, Proteintech), anti-Aromatase (#ab18995, Abcam), anti-ESR2 (#14007-1-AP, Proteintech), anti-eIF4E (#2067, CST), anti-eIF3e (#A3431, Abclonal), anti-eIF3i (#11287-1-AP, Proteintech). Secondary antibodies include HRP-conjugated Goat Anti-rabbit IgG (#SA00001-2, Proteintech) and HRP-conjugated Goat Anti-Mouse IgG (#SA00001-1, Proteintech). Antibodies for immunoprecipitation include anti-METTL3 (#ab195352, Abcam), Normal Rabbit IgG (#2729, CST) and HRP IP Detection Reagent (#21,230, Thermo). TRITC-Phalloidin (#P1951) was purchased from Sigma (St. Louis, MO, USA). 4EGI-1 (#HY-19,831) and STM2457 (#HY-134,836) were purchased from MCE (Shanghai, China).

### Bioinformatics analysis

Bioinformatics analysis was performed using public databases (TCGA, CPTAC) and published datasets to further explore the expression of METTL3, CYP19A1, ESR2 in NSCLC samples. The differentially expressed genes were screened using the R package “limma”, and those with absolute values of log of fold change (logFC) > 1 and *P* < 0.05 were considered as differentially expressed genes. The RNA sequencing and polysome profiling data from Choe et al. [11] was applied to identified the genes with changes in translation efficiency regulated by METTL3. KEGG enrichment analysis was performed using the R package clusterProfiler on 4269 genes encoding mRNAs with significantly reduced translation efficiency.

## Results

### High expression of METTL3 correlates with NSCLC metastasis and is associated with poor prognosis

**Fig. 1:**
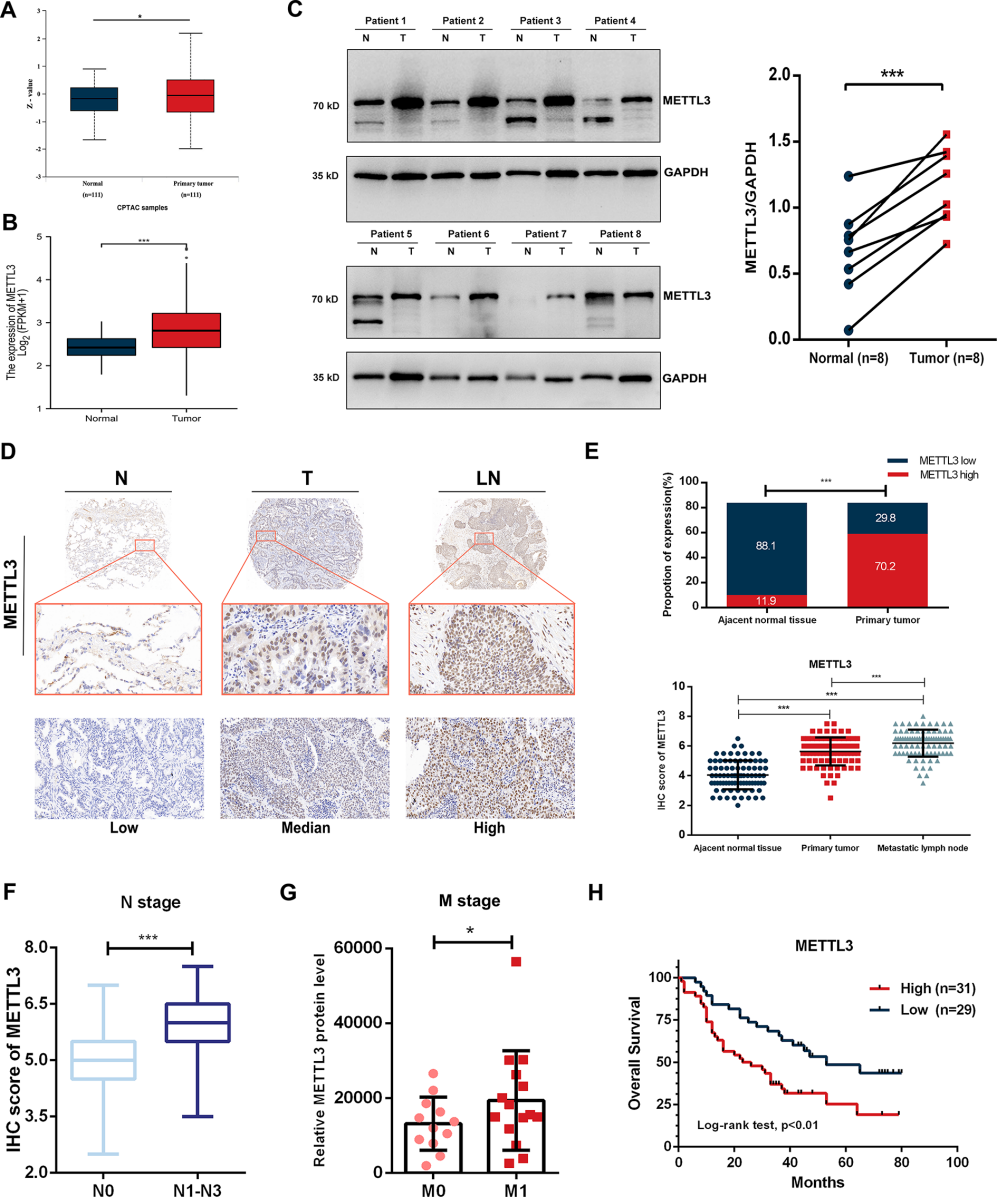
METTL3 is highly expressed in primary lesions and metastatic lymph nodes of NSCLC, indicating a poor prognosis. **(A)** The CPTAC database indicates high expression of the METTL3 protein in lung adenocarcinoma tissues. **(B)** The TCGA database indicates high expression of METTL3 mRNA in NSCLC. **(C)** Western blot analysis indicates high expression of METTL3 in tumour tissues from 8 NSCLC patients. The right panel displays the quantification results of the left panel. **(D)** IHC staining of a tissue microarray from 96 patients indicates high expression of METTL3 in primary lesions and metastatic lymph nodes of NSCLC. **(E)** Quantification results of IHC staining in tissue microarrays. **(F)** METTL3 expression of primary tumours in N0 stage patients is significantly lower than that in N1-N3 stage patients. **(G)** DIA-MS results from late-stage NSCLC revealed that METTL3 expression of primary tumours in M0 stage patients is significantly lower than that in M1 stage patients. **(H)** Survival analysis demonstrates the relationship between METTL3 expression in tissue microarrays and the prognosis of NSCLC patients, with high expression of METTL3 suggesting poor prognosis

To explore the role of METTL3 in NSCLC, we first analyzed The Cancer Genome Atlas (TCGA) and the Clinical Proteomic Tumour Analysis Consortium (CPTAC) databases. The results revealed that both METTL3 mRNA and protein expression levels were elevated in primary NSCLC tissues compared with paired normal lung tissues (Fig. 1A-B). To verify these findings, we examined METTL3 expression in our cohort of NSCLC samples. Western blotting analysis demonstrated high METTL3 protein expression in NSCLC tissues compared with paired normal lung tissues (*n* = 8, Fig. 1C). Furthermore, immunohistochemistry (IHC) staining was performed on tissue microarrays of paired clinical specimens collected from 84 patients in our cohort. The results demonstrated a significant increase in METTL3 expression in primary NSCLC, with the highest expression observed in metastatic lymph nodes compared to adjacent normal tissues (Fig. 1D-E). Furthermore, we categorized the patients into two groups: the N0 group consisting of individuals without lymph node metastasis, and the N1-N3 group composed of patients with lymph node metastasis. By comparing the METTL3 expression in the primary tumour between these groups, we observed a significant association between high METTL3 expression and a more advanced lymph node stage (Fig. 1F; Table 1, Table S1). Additionally, to analyze the correlation between METTL3 expression and distant metastasis, we utilized the data independent acquisition-mass spectrometry (DIA-MS) data from a late-stage NSCLC cohort conducted by Lehtiö et al. [24] Comparing the METTL3 expression in biopsy samples from 12 M0 stage patients and 15 M1 stage patients, we discovered that patients with distant metastasis exhibited higher levels of METTL3 compared to those without distant metastasis (Fig. 1G, Table S2). Finally, we assessed METTL3 expression and its association with the prognosis of the 84 NSCLC patients, revealing that a high METTL3 expression level is correlated with poor overall survival (OS) (Fig. 1H). We also analyzed the correlation between METTL3 expression and other clinical characteristics, which are summarized in Table 1. Taken together, these results suggest the essential function of METTL3 in NSCLC progression and metastasis.

**Table 1:** Correlation between METTL3 and CYP19A1 expression with clinical characteristics

		METTL3 expression				CYP19A1 expression						
Characteristics	Total (*n* = 84)	Low (*n* = 25)	High (*n* = 59)	χ2	P value	Low (*n* = 26)	High (*n* = 58)		χ2		P value	
Gender Male Female				0.04314	0.8355				1.951		0.1624	
	66	20	46			18	48					
	18	5	13			8	10					
Age at surgery ≥ 60 ＜ 60				0.7921	0.3735				0.1442		0.7042	
	33	8	25			11	22					
	51	17	34			15	36					
TNM stage				0.2847	0.5936				14.44		**0.0001**	
I-II	30	10	20			17	13					
III	54	15	39			9	45					
TNM stage: T				0.1452	0.7032				6.641		**0.0100**	
T1-T2	58	18	40			23	35					
T3-T4	26	7	19			3	23					
TNM stage: N				7.999	**0.0047**				10.36		**0.0013**	
N0	20	11	9			12	8					
N1-3	64	14	50			14	50					
Tumor histology				0.2739	0.6007				2.553		0.1101	
SQC	44	12	32			17	27					
ADC	40	13	27			9	31					

*Note P* values were derived from the χ2 test; *P* values in bold font indicate statistically significant differences. SQC represents squamous cell carcinoma and ADC represents adenocarcinoma. TNM staging is based on the eighth edition of UICC/AJCC lung cancer stage classification (2017)

### METTL3 promotes migration, invasion and invadopodia formation in NSCLC cells

**Fig. 2:**
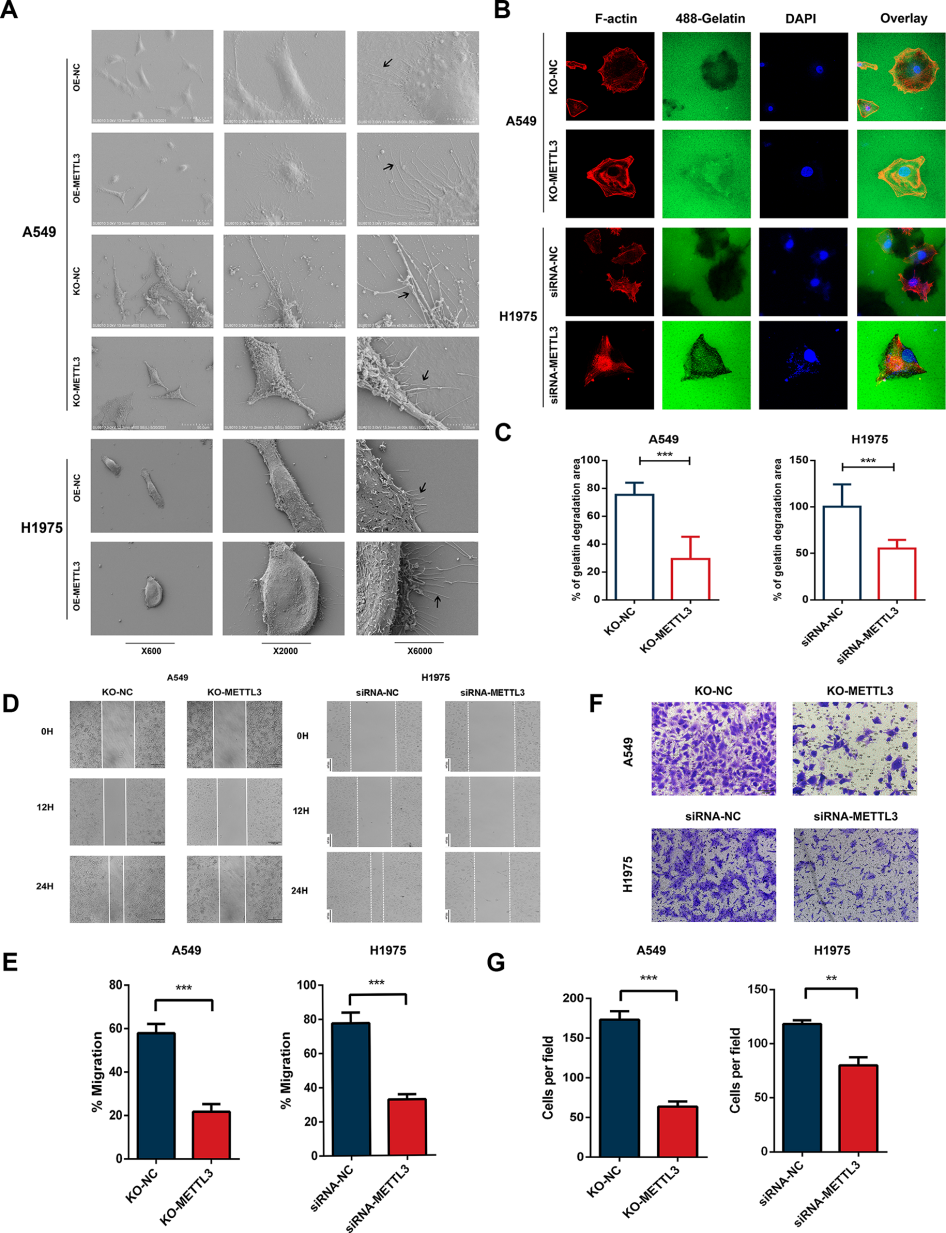
METTL3 promotes migration, invasion and invadopodia formation in NSCLC cells. **(A)** Scanning electron microscopy showed that overexpression of METTL3 in A549 and H1975 cells resulted in increased and elongated pseudopodia, while knockout of METTL3 led to decreased and shortened pseudopodia in the cells. **(B**-**C)** In the fluorescence matrix degradation experiment, the depth and extent of extracellular matrix degradation were significantly reduced when METTL3 was knocked out in A549 cells using CRISPR‒Cas9 technology. Furthermore, the depth and extent of extracellular matrix degradation were reduced in H1975 cells after METTL3 knockdown. **(D**-**E)** The wound healing experiment after METTL3 knockout demonstrated a significant decrease in cell migration ability in A549 cells. After transfection with siRNA targeting METTL3, there is a significant decrease in the migration ability of H1975 cells. **(F**-**G)** Transwell invasion experiments after METTL3 knockout showed a significant decrease in cell invasion ability of A549 cells. After transfection with siRNA targeting METTL3, there is a significant decrease in the invasion ability of H1975 cells

Through the analysis of clinical data, we hypothesized that high METTL3 expression might be associated with the progression and metastasis of NSCLC. To further explore this, we conducted in vitro experiments using siRNA and plasmid transfection to knockdown or overexpress METTL3 in A549 and H1975 cells. Additionally, we established a CRISPR system to deplete METTL3 (METTL3 KO) in A549 cells. As invasive protrusions, known as invadopodia, play an important role in supporting tumour cell invasion and dissemination [25], we first utilized scanning electron microscopy to examine the cell morphology of A549 and H1975 cells (Fig. 2A). The results demonstrated that METTL3 KO led to a decrease in invadopodia formation, while METTL3 overexpression exerted the opposite effect. Using the Gelatin Invadopodia Assay, we then validated that METTL3 promotes the ability of NSCLC cells to form invadopodia and degrade the extracellular matrix (Fig. 2B-C). Furthermore, we investigated the effect of METTL3 on cell migration and invasion using Transwell (Fig. 2D-E) and wound healing assays (Fig. 2F-G). The knockdown of METTL3 in H1975 cells and METTL3 KO in A549 cells both inhibited cell migration and invasion. In summary, through our experiments, we observed the impact of METTL3 on various aspects of NSCLC progression and metastasis, supporting the notion that METTL3 plays a significant role in these processes.

### METTL3 depletion inhibits metastatic potential of lung cancer cells in vivo

**Fig. 3:**
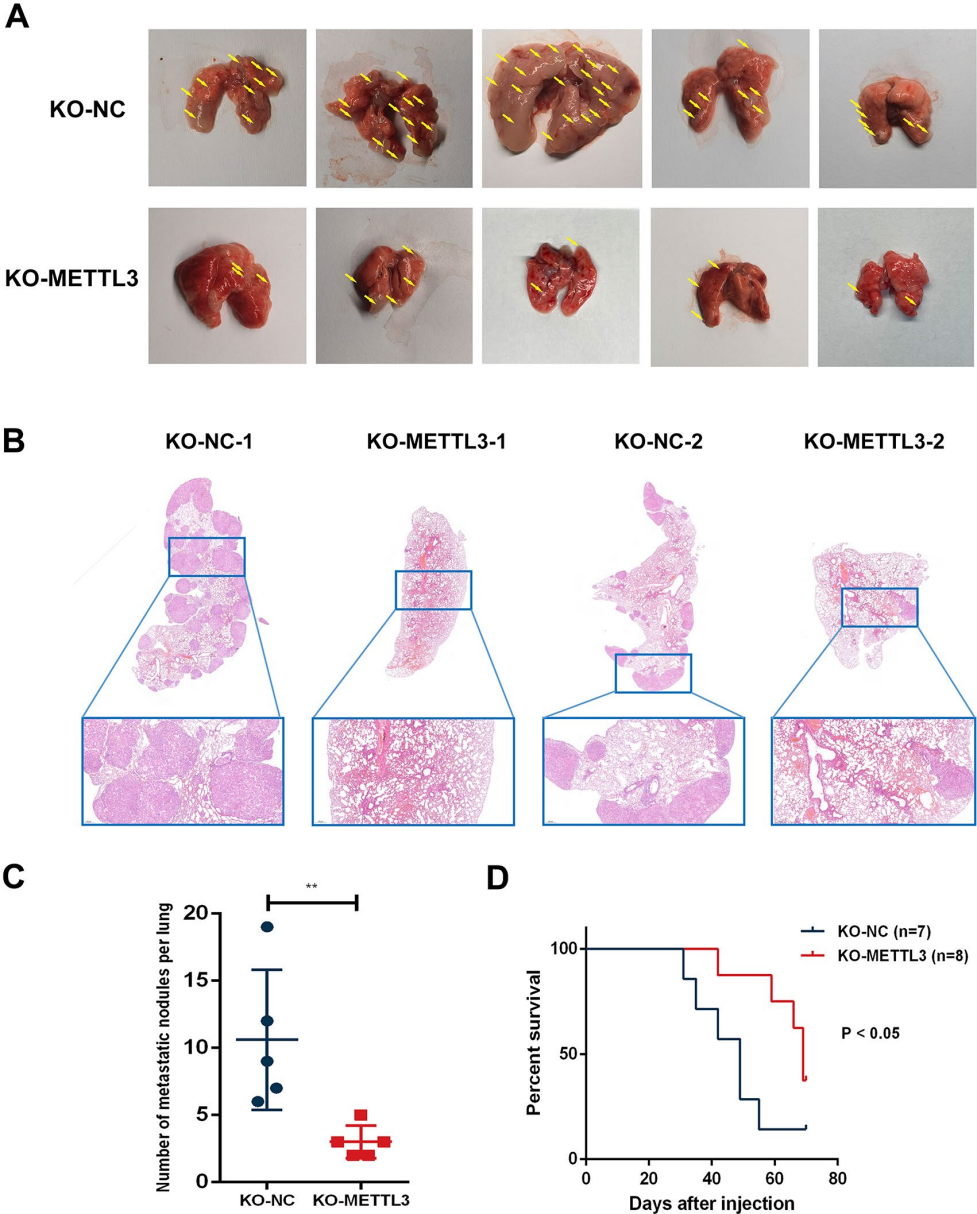
METTL3 depletion inhibits metastatic potential of lung cancer cells in vivo. **(A)** Macroscopic image of mouse lungs injected with NC-METTL3 cells and KO-METTL3 cells via the tail vein. The NC-METTL3 group exhibited a significantly higher number of metastatic tumours than the KO-METTL3 group. **(B)** Histological sections of mouse lungs with HE staining. The NC-METTL3 group showed a significantly higher number of metastatic noduless than the KO-METTL3 group. **(C)** Quantification of lung metastatic nodules in mice from the NC-METTL3 group and the KO-METTL3 group. The NC-METTL3 group demonstrated significantly more lung metastatic tumours than the KO-METTL3 group. **(D)** Kaplan‒Meier survival curves for the KO-NC group and KO-METTL3 group

To further investigate the effects of METTL3 on NSCLC cells in vivo, we established a lung metastasis model by injecting NSCLC cells into the tail veins of nude mice [26, 27]. After a six-week modelling period, the mice were euthanized, and their lungs were extracted and assessed. As observed macroscopically, METTL3 KO significantly reduced the number of tumor lesions in the lungs (Fig. 3A). HE staining and statistical analysis revealed that METTL3 KO significantly inhibited the ability of tumour cells to colonize the lungs of injected mice (Fig. 3B-C). Additionally, survival analysis using this lung metastasis model demonstrated that METTL3 depletion promoted OS (Fig. 3D). These findings suggest that METTL3 can enhance the lung metastatic potential of NSCLC cells in vivo.

### CYP19A1 is a potential target of METTL3-mediated translation regulation

**Fig. 4:**
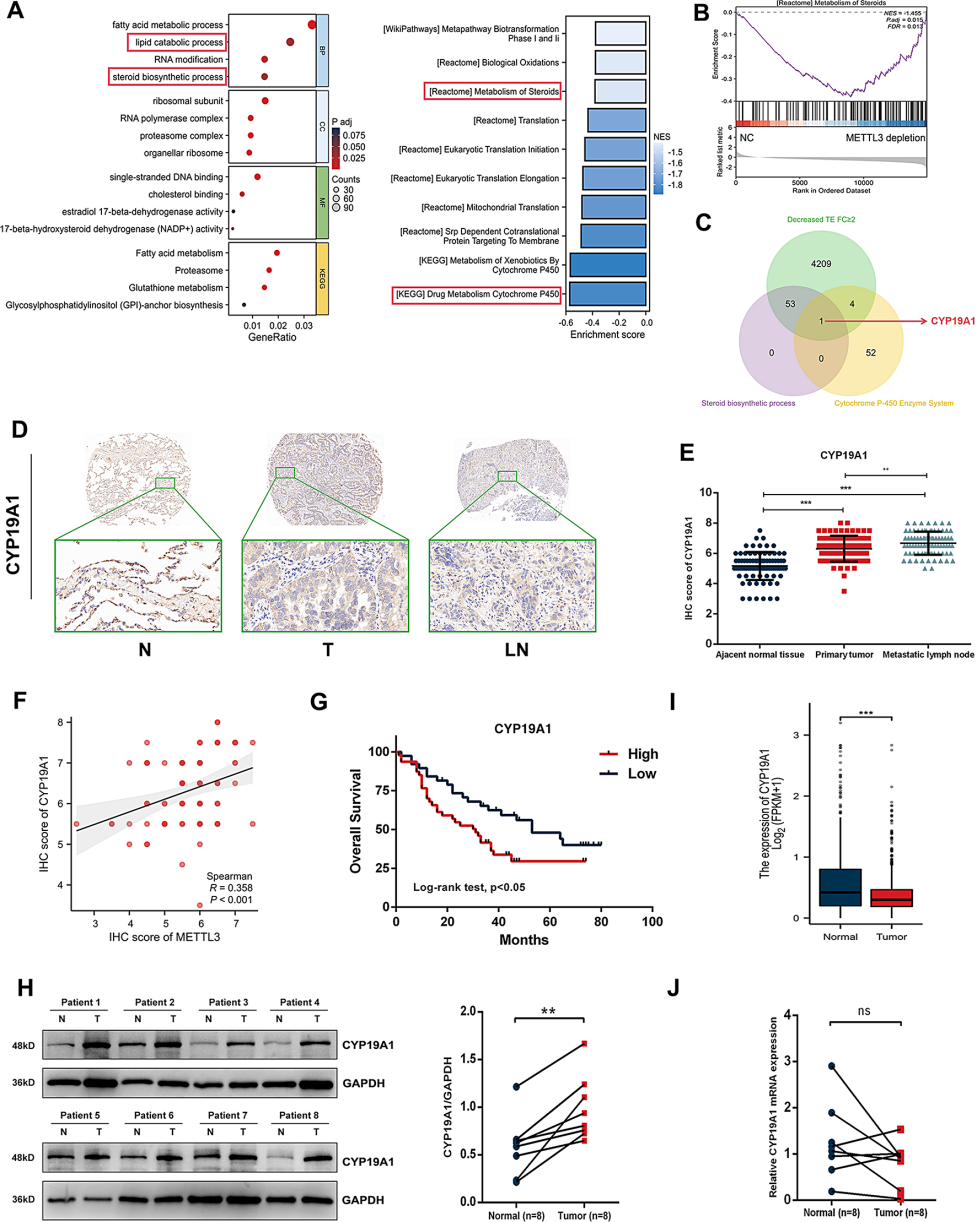
CYP19A1 is the potential target of METTL3-mediated translation regulation. **(A**-**B)** GO-KEGG analysis and GSEA of genes with decreased translation efficiency after METTL3 knockout indicated the association between METTL3 and the steroid signalling pathway. **(C)** Genes downregulated in translation efficiency after METTL3 depletion were crossed with genes involved in steroid biosynthetic processes and the cytochrome P450 enzyme system to obtain CYP19A1. **(D**-**E)** IHC staining analysis of patient tissue microarray showed that compared to adjacent tissues, CYP19A1 protein was highly expressed in both the primary lesions and metastatic lymph nodes of NSCLC. **(F)** The results of IHC analysis indicated that CYP19A1 expression was positively correlated with METTL3 expression in NSCLC. **(G)** Survival analysis of the tissue microarray indicated that high expression of CYP19A1 suggests poor prognosis of NSCLC patients. **(H)** Western blotting analysis validated that the CYP19A1 protein is highly expressed in NSCLC tissues compared with paired normal lung tissues. **(I)** Data from the TCGA database indicated that CYP19A1 mRNA expression in NSCLC is lower than that in normal lung tissue. **(J)** RT‒qPCR indicated that CYP19A1 mRNA expression in NSCLC is not higher than that in paired normal lung tissue

METTL3 is involved in important processes in tumours by mediating m^6^A modifications [28]. We analyzed the genes with decreased translation efficiency after METTL3 depletion from the data of Chole et al. [11]. and found that the genes with decreased translation efficiency were enriched in the steroid biosynthetic process and metabolism of cytochrome P450 pathway through GO/KEGG and GSEA, suggesting that there may be some association between METTL3 and the steroid signalling pathway (Fig. 4A-B). Subsequently, we crossed the genes downregulated in translation efficiency after METTL3 depletion with genes involved in steroid biosynthetic processes and the cytochrome P450 enzyme system. We noted that among the overlapping genes, the critical oestrogen synthesis gene CYP19A1 was revealed (Fig. 4C, Table S3). CYP19A1 acts as a rate-limiting enzyme in oestrogen biosynthesis and its role in the final step of oestrogen biosynthesis is crutial for the activation of the E2/ERβ signalling pathway. Moreover, some studies have reported that the expression of CYP19A1 in NSCLC has a high positive correlation with ERβ and can activate the E2/ERβ signalling pathway by promoting oestrogen production, leading to the progression of NSCLC; thus, CYP19A1 can be used as a predictor of patient survival [29, 30].

### CYP19A1 protein but not mRNA is highly expressed in NSCLC tissues

Therefore, we first analyzed the expression and prognostic value of CYP19A1 in NSCLC first. By performing IHC staining of tissue microarrays of paired clinical specimens collected from 84 patients, our observations revealed a noteworthy elevation in CYP19A1 expression levels in primary NSCLC, with the highest expression level observed in metastatic lymph nodes compared to adjacent normal tissues (Fig. 4D-E). Moreover, data from tissue microarrays also revealed that CYP19A1 expression is positively correlated with METTL3 expression in primary NSCLC (Fig. 4F). The correlation between CYP19A1 expression and other clinical characteristics was also analyzed (Table 1). We found that high CYP19A1 expression levels in primary tumours were significantly correlated with a higher TNM stage and T/N stage, suggesting that CYP19A1 might play an indispensable role in NSCLC metastasis. Furthermore, we found that a high CYP19A1 expression level is correlated with poor overall survival (OS) by analysing data from our cohort (Fig. 4G). Western blotting analysis also validated that the CYP19A1 protein is highly expressed in NSCLC tissues compared with paired normal lung tissues (*n* = 8, Fig. 4H).

Interestingly, data from the TCGA database revealed that CYP19A1 mRNA is not highly expressed in NSCLC and that its expression level is even lower than that in normal lung tissue (Fig. 4I). We validated this finding by performing RT-qPCR and found that the CYP19A1 mRNA expression level was not higher than that in paired normal lung tissue (Fig. 4J). The above results support the sequencing data indicating that CYP19A1 might be translationally regulated by METTL3, resulting in its elevated protein expression level but not its mRNA expression level in primary NSCLC.

### METTL3 mediates m^6^A modification of CYP19A1 and promotes the synthesis of E2

**Fig. 5:**
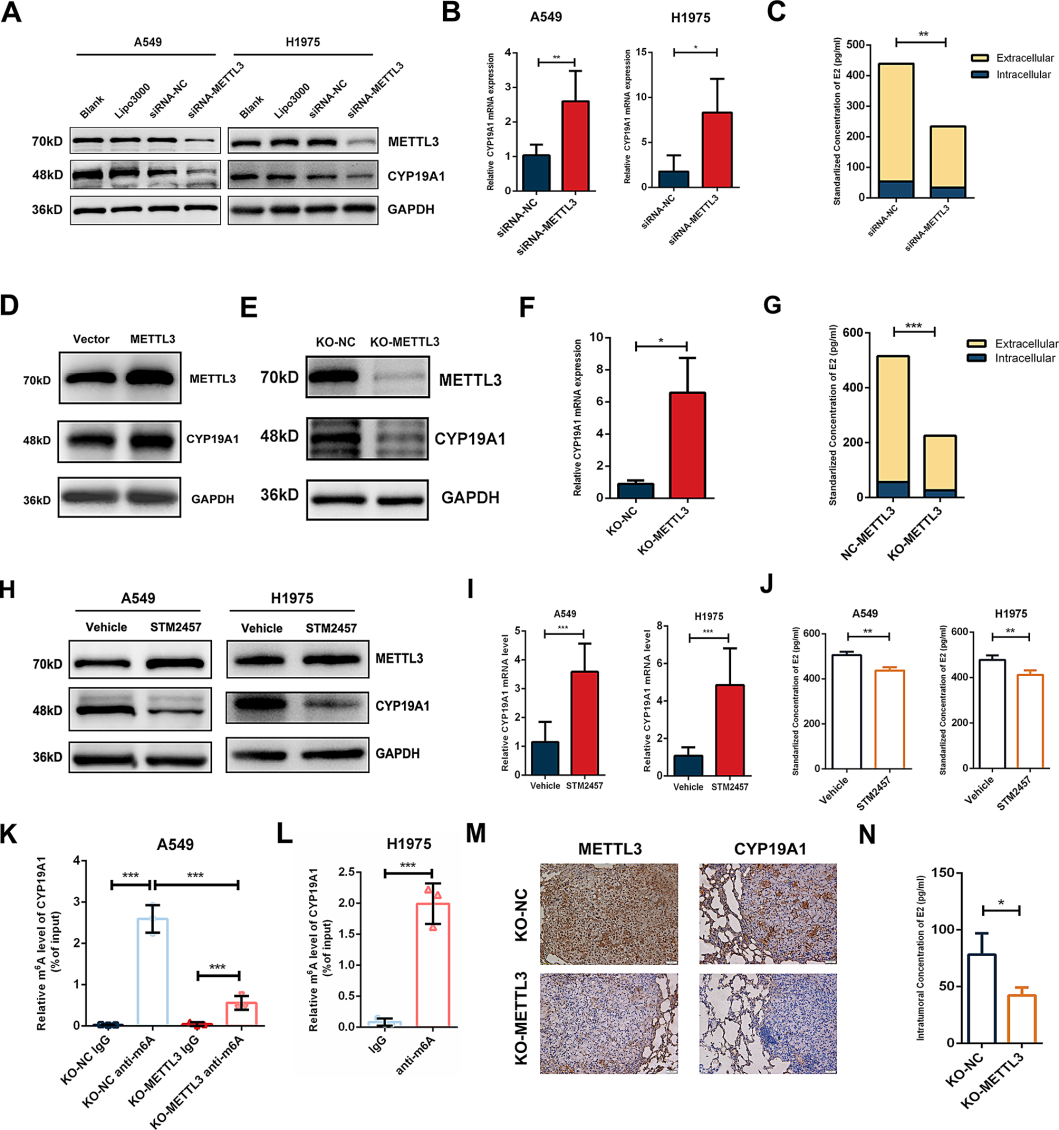
METTL3 mediates m^6^A modification of CYP19A1 and promotes the synthesis of E2. **(A)** WB experiments demonstrated that knockdown of METTL3 using siRNA significantly reduced the protein expression of CYP19A1. **(B)** PCR experiments showed an upwards trend in the mRNA expression of CYP19A1 following siRNA knockdown of METTL3. **(C)** ELISA revealed a decrease in intracellular and extracellular levels of E2 after siRNA knockdown of METTL3. **(D)** CYP19A1 is upregulated after transfection of plasmids for METTL3 overexpression. **(E)** Protein expression of CYP19A1 is significantly downregulated after CRISPR‒Cas9 knockout of METTL3. **(F)** PCR indicated an upwards trend in the mRNA expression of CYP19A1 following CRISPR‒Cas9 knockout of METTL3. **(G)** ELISA demonstrated a significant decrease in intracellular and extracellular levels of E2 after CRISPR‒Cas9 knockout of METTL3. **(H)** Treatment with 5 µM STM2457 for 3 days led to a significant decrease in the protein level of CYP19A1. **(I)** Treatment with 5 µM STM2457 for 3 days led to a significant increase in the mRNA level of CYP19A1. **(J)** Treatment with 5 µM STM2457 for 3 days led to a significant reduction in E2 levels. **(K)** MeRIP-qPCR revealed that CYP19A1 mRNA was enriched in m^6^A modification and the relative levels of m^6^A whin CYP19A1 were significantly downregulated after METTL3 knockout in A549 cells. **(L)** MeRIP-qPCR revealed that CYP19A1 mRNA was enriched in m^6^A modification in H1975 cells. **(M**-**N)** CYP19A1 protein expression and intratumoural E2 concentration in lung metastasis of KO-METTL3 tumours are significantly downregulated

In clinical specimens, we found that the level of CYP19A1 protein but not mRNA was abnormally highly expressed in NSCLC tissues. To explore the regulatory role played by METTL3, we regulated the expression of METTL3 in NSCLC cell lines and examined the expression of CYP19A1 and the production of E2. We found that transient knockdown of METTL3 resulted in a decrease in the protein expression level of CYP19A1 in A549 and H1975 cell, with a corresponding decrease in the intra- and extracellular E2 levels; however, there was an inconsistent increase in the mRNA levels of CYP19A1 after METTL3 knockdown (Fig. 5A-C). Overexpression of METTL3 through plasmid transfection resulted in the upregulation of CYP19A1 protein expression (Fig. 5D). After stable knockout of METTL3 in A549 cells line, we compared the KO-NC group with the KO-METTL3 group, and found that knockdown of METTL3 resulted in a decrease in the protein expression levels of CYP19A1 and ERβ as well as a decrease in the intra- and extracellular production of E2. Similar to the effect of transient transfection, knockout of METTL3 also resulted in elevated CYP19A1 mRNA levels (Fig. 5E-G).

To further determine METTL3’s function in regulating the expression of CYP19A1, we introduced a small-molecule inhibitor of METTL3, namely, STM2457. STM2457 specifically inhibits the m^6^A catalytic activity of METTL3 without disrupting the structure of the METTL3-METTL14 methyltransferase complex [31]. A549 and H1975 cells were divided into the STM2457 (5 µM for 3 days) treatment group and the control group (DMSO for 3 days). By western blot analysis, we found that the protein level of CYP19A1 was significantly downregulated after STM2457 intervention compared with the control group (Fig. 5H); by RT‒qPCR, we found that the mRNA level of CYP19A1 was significantly increased after STM2457 intervention compared with the control group (Fig. 5I). ELISA analysis revealed that STM2457 treatment also decreased the extracellular E2 concentration (Fig. 5J). The above experiments suggested that STM2457 can block the protein synthesis of CYP19A1 by inhibiting METTL3-mediated m^6^A modification.

To validate the crosstalk between CYP19A1 and METTL3 relies on m^6^A modification, we initially utilized the online tool SRAMP (http://www.cuilab.cn/sramp)[23]. This tool predicted an m^6^A modification site with extremely high confidence on the 3’ UTR of CYP19A1 mRNA. Subsequently, we performed MeRIP experiments using an m^6^A-specific antibody, followed by RT-qPCR with precise primers targeting the identified m^6^A modification site. Interestingly, we observed a significant reduction in m^6^A-modified CYP19A1 levels in KO-METTL3 cells compared to KO-NC cells (Fig. 5K). Moreover, in line with our findings in A549 cells, we successfully validated the enrichment of the same m^6^A modification site in CYP19A1 mRNA of H1975 cells (Fig. 5L). In addition, we examined CYP19A1 protein expression and the intratumoural E2 concentration in lung lesions of KO-NC and KO-METTL3 tumours in the tail-vein injection model, and the results showed that CYP19A1 protein and E2 levels were significantly decreased after METTL3 knockout (Fig. 5M-N).

In conclusion, inhibition of METTL3 in NSCLC cell lines downregulated CYP19A1 protein expression and E2 production and conversely resulted in an increase in CYP19A1 mRNA expression levels. This demonstrates that METTL3 is involved in the protein synthesis of CYP19A1 mRNA through m^6^A modification.

### Translation of CYP19A1 mRNA is dependent on the regulation of METTL3

**Fig. 6:**
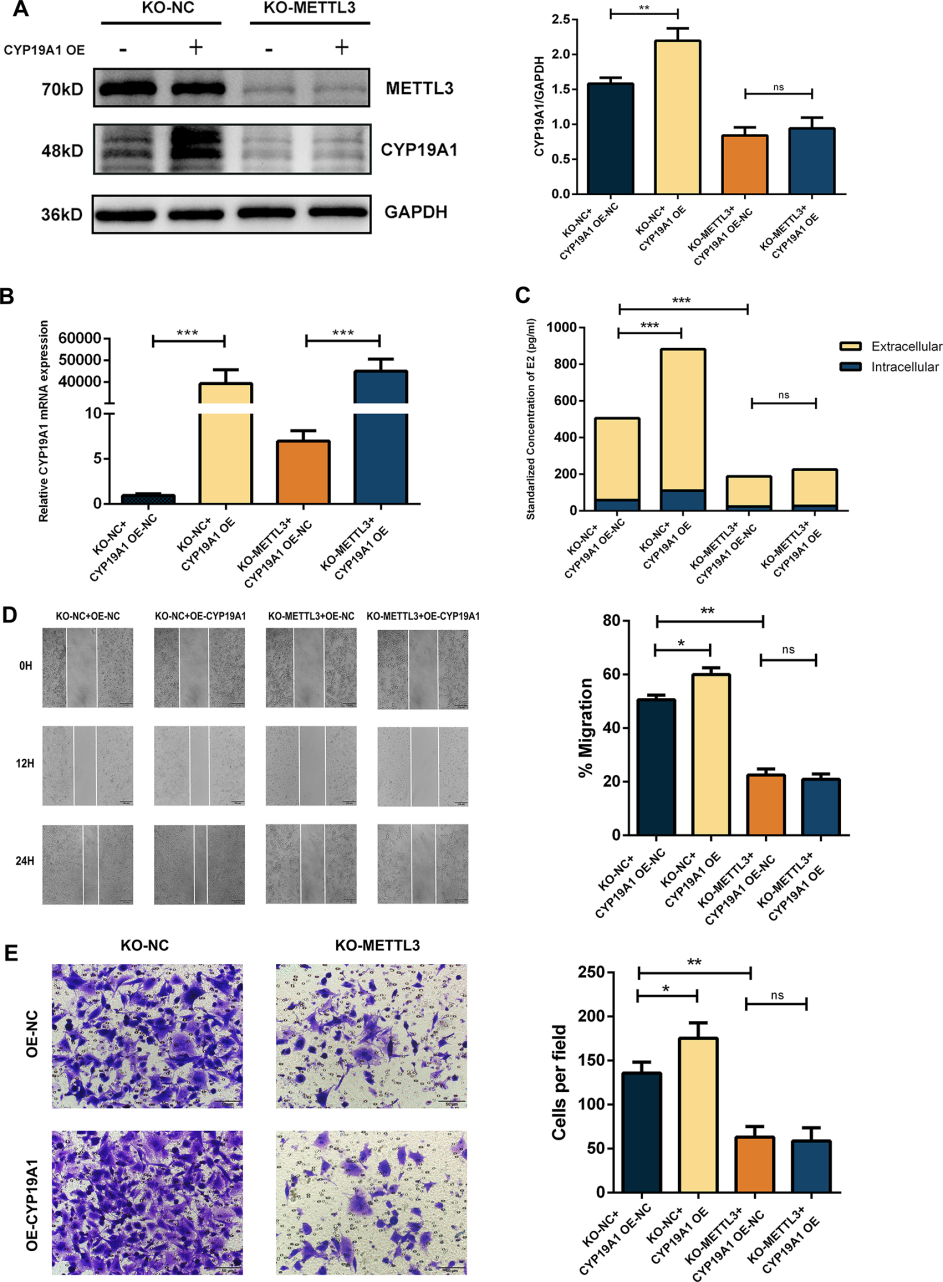
Translation of CYP19A1 mRNA is dependent on the regulation of METTL3. **(A)** In KO-METTL3 cells, the transient transfection of plasmids was used to upregulate the mRNA levels of CYP19A1, while METTL3 deficiency abolished its effect on CYP19A1 protein expression. However, in the KO-NC group, transient overexpression of CYP19A1 mRNA successfully led to overexpressed CYP19A1 protein. **(B)** PCR results showed that both the KO-METTL3 and KO-NC groups transfected with plasmids transiently overexpressed CYP19A1 mRNA. **(C)** In the KO-NC group, transient overexpression of CYP19A1 mRNA led to upregulation of E2 levels, while in the KO-METTL3 group, METTL3 deficiency decreased intracellular and extracellular E2 secretion. **(D)** Wound healing experiments showed that in the KO-NC group, transient upregulation of CYP19A1 mRNA significantly enhanced cell migration ability. However, transient upregulation of CYP19A1 mRNA did not significantly alter the cell migration ability under METTL3 deficiency. **(E)** Transwell invasion assay showed that in the KO-NC group, transient upregulation of CYP19A1 mRNA significantly enhanced cell invasion ability. However, transient upregulation of CYP19A1 mRNA did not significantly alter the cell invasion ability under METTL3 deficiency

To demonstrate that METTL3 plays an essential role in the translation of CYP19A1 mRNA, we overexpressed CYP19A1 mRNA by transient transfection in KO-METTL3 cells, detected CYP19A1 protein expression and E2 production, and assessed migration and invasion potential. The 4 groups were KO-NC cells transfected with negative control plasmid (KO-NC + OE-NC), KO-NC cells transfected with CYP19A1 overexpressing plasmid (KO-NC + OE-CYP19A1), KO-METTL3 cells transfected with negative control plasmid (KO-METTL3 + OE-NC), and KO-METTL3 cells transfected with CYP19A1 overexpressing plasmid (KO-METTL3 + OE-CYP19A1). By RT‒PCR, we observed that CYP19A1 mRNA was successfully overexpressed in both KO-NC cells and KO-METTL3 cells (Fig. 6B). Western blot analysis revealed that in KO-NC cells, the overexpression of CYP19A1 mRNA led to a significant increase in its protein level, while in KO-METTL3 cells, the overexpression of CYP19A1 mRNA did not cause an increase in its protein level (Fig. 6A). The ELISA results were consistent with the WB results. In KO-NC cells, overexpression of CYP19A1 mRNA induced an increase in intra- and extracellular E2 concentrations by upregulating its protein expression, whereas in KO-METTL3 cells, overexpression of CYP19A1 mRNA did not cause an increase in the E2 concentration (Fig. 6C).

By the same grouping, we found that in KO-NC cells, overexpression of CYP19A1 mRNA led to an increase in the migration of cells by the wound healing assay and the invasion of cells by the Transwell invasion assay. In contrast, in KO-METTL3 cells, overexpression of CYP19A1 mRNA failed to rescue the migration and invasion abilities of NSCLC cells (Fig. 6D-E).

In summary, these data revealed that CYP19A1 is an essential downstream target of METTL3 and further supported that CYP19A1 protein expression the above rescue experiments further confirmed that translation of CYP19A1 mRNA is dependent on the existence of METTL3.

### METTL3 binds to CYP19A1 mRNA and interacts with eIFs to regulate the translation efficiency of CYP19A1

**Fig. 7:**
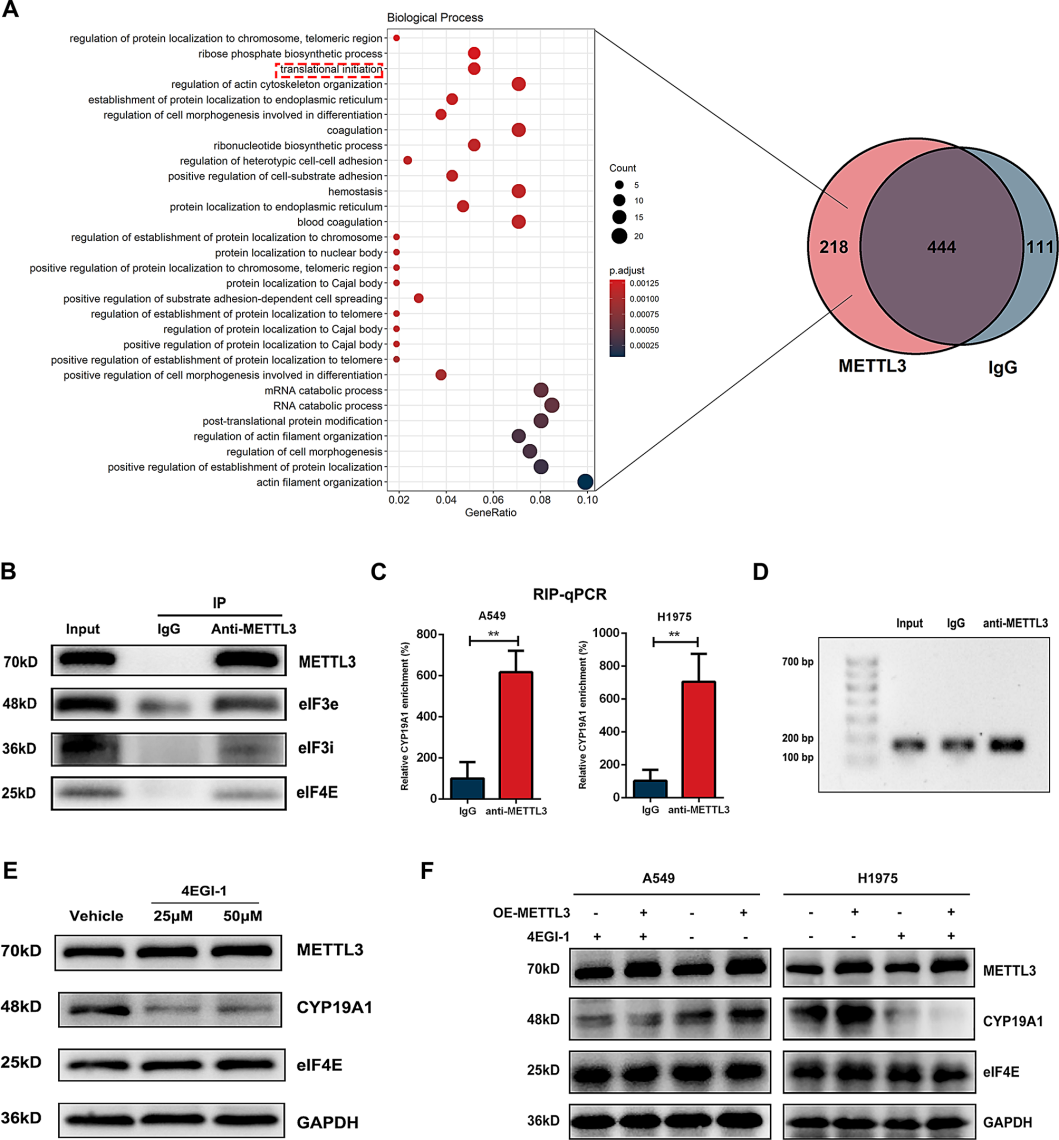
METTL3 binds to CYP19A1 mRNA and interact with eIFs to regulates translation efficiency of CYP19A1. **(A)** Immunoprecipitation using anti-METTL3 followed by mass spectrometry analysis was performed to identify proteins that interact with METTL3. A total of 218 proteins were identified as specific binding proteins of METTL3, which are presented in a Venn diagram. GO analysis of the 218 proteins revealed enrichment in the translation initiation process. **(B)** Co-IP to confirm the interaction between METTL3 and translation initiation molecules identified by mass spectrometry, including eIF3e, eIF3i, and eIF4E. **(C**-**D)** RNA immunoprecipitation using anti-METTL3 antibody followed by qPCR validation showing direct binding of METTL3 to CYP19A1 mRNA in A549 and H1975 cells. Agarose gel electrophoresis of qPCR samples confirmed the enrichment of CYP19A1 mRNA in the anti-METTL3 group, confirming the binding between METTL3 and CYP19A1 mRNA. **(E)** Treatment with the eIF4E inhibitor 4EGI-1 significantly downregulated the protein expression of CYP19A1. **(F)** Administration of 4EGI-1 abolished the effect of METTL3 overexpression in promoting CYP19A1 expression

To study the molecular mechanism underlying METTL3-mediated translation regulation of CYP19A1 mRNA, we first clarified the proteins interacting with METTL3 in NSCLC cells. We obtained proteins that interact with METTL3 in A549 cells by immunoprecipitation and identified these proteins by mass spectrometry. The Venn diagram (Fig. 7A) indicates that 662 proteins were pulled down by the METTL3 antibody, 555 proteins were pulled down by IgG, and 218 proteins were specifically pulled down by the METTL3 antibody but not IgG. GO analysis of these 218 proteins revealed that they could be enriched in the translation initiation pathway (Fig. 7A, Table S4). Among the pathways, there are three important eukaryotic translation initiation factors, eIF3e, eIF3i and eIF4E. We then verified this result by coimmunoprecipitation and clarified that METTL3 is able to bind to eIF4E, eIF3e, and eIF3i (Fig. 7B).

After determining that METTL3 can bind to translation initiation molecules, we initially clarified the key role played by METTL3 in translation regulation in NSCLC. To further confirm that METTL3 can directly participate in the translation of CYP19A1, we collected the mRNA bound by METTL3 in A549 and H1975 by RNA immunoprecipitation assay. After performing reverse transcription and RT-qPCR experiments, our findings indicated that METTL3 is capable of binding to CYP19A1 mRNA in A549 and H1975 cells (Fig. 7C). Subsequently, we used the products of RT‒qPCR for DNA agarose gel electrophoresis analysis and found that the anti-METTL3 group, which was immunoprecipitated using the antibody to METTL3, showed higher enrichment of CYP19A1 mRNA than both the input group and the IgG group (Fig. 7D, Figure S3). The above results indicated that METTL3 protein could directly bind to CYP19A1 mRNA.

### eIF4E is essential for METTL3-mediated CYP19A1 translation

Through the above experiments, we clarified that METTL3 can interact with translation initiation factors such as eIF4E and that METTL3 can directly bind to CYP19A1 mRNA and thus participate in the translational regulation of CYP19A1. However, the importance of eIFs in the process of CYP19A1 translation remains to be clarified. Since eIF4E is a key molecule in the translation initiation complex and there is a well-established inhibitor of eIF4E, we introduced the eIF4E/eIF4G costimulatory inhibitor 4EGI-1 to block the cap-binding protein-dependent mRNA translation process [32]. We divided A549 cells into a vehicle group, 25 µM 4EGI-1 group, and 50 µM 4EGI-1 group and performed western blotting after 1 day of intervention. The results showed that 4EGI-1 had no effect on either the expression of eIF4E itself or the expression of METTL3 but that both 25 µM and 50 µM 4EGI-1 led to a significant decrease in CYP19A1 protein expression (Fig. 7E).

Furthermore, we overexpressed METTL3 in the A549 and H1975 cell lines and found that administration of 4EGI-1 significantly downregulated and abolished the effect of METTL3 in promoting CYP19A1 expression (Fig. 7F). Together, we concluded that METTL3 promotes CYP19A1 protein expression through eIF4E-mediated translation.

## Discussion

Tumour metastasis, a prominent topic in lung cancer research, involves complex and diverse processes. METTL3, a key protein closely associated with tumour progression and recently discovered, requires further exploration regarding its functional role in the invasion and metastasis of NSCLC. In this study, we analyzed the expression of METTL3 in the public databases and validated it through western blotting and immunohistochemical analysis of clinical specimens from NSCLC patients. The results showed that there are high expression levels of METTL3 in NSCLC, and it is highly correlated with metastasis. Moreover, in conjunction with clinical data, we determined that high expression levels of METTL3 in NSCLC indicate poor prognosis, consistent with reported findings in the literature [33–35].

To elucidate the impact of METTL3 on invasion and migration of lung cancer cells and its role in facilitating the metastatic potential of solid tumour cells, we employed various techniques to modulate METTL3 expression in A549 and H1975 NSCLC cells. Electron microscopy observations demonstrated that morphologically, METTL3 promotes the formation of pseudopodia in NSCLC cells, enabling their transition towards a more invasive phenotype. Fluorescence-based matrix degradation assays revealed that METTL3 enhances the extension of pseudopodia through cytoskeletal proteins, leading to the degradation of the extracellular matrix and laying the foundation for the invasion of basal membranes during the metastatic process. Scratch wound healing assays demonstrated that METTL3 enhances the migration of NSCLC cells, supporting its potential role in promoting lung cancer metastasis. Finally, in Transwell invasion assays simulating cell invasion through the extracellular matrix, METTL3 demonstrated the ability to promote invasion of NSCLC cells, thus suggesting its potential to facilitate cancer cell penetration into the circulation by traversing the extracellular matrix, leading to metastasis. Additionally, to preliminarily investigate the pharmacological effects of STM2457 in NSCLC, we observed a significant inhibition of cell migration and invasion after drug intervention in A549 and H1975 cells in in vitro experiments. In in vivo experiments, we also provided evidence for the promotion of metastasis of NSCLC by METTL3.

Through secondary mining of transcriptomic sequencing data from HeLa cells in the literature, we identified CYP19A1 as a downstream target of METTL3 in the oestrogen signalling pathway. Subsequently, we confirmed significant inconsistencies in the expression of CYP19A1 at the mRNA and protein levels in clinical specimens of NSCLC, indicating abnormal translation. In cell experiments, by knocking down or knocking out METTL3 expression in A549 and H1975 cells or using the METTL3 small-molecule inhibitor STM2457, we observed a significant decrease in CYP19A1 protein expression and a corresponding reduction in E2 production. Conversely, in CYP19A1-overexpressing cells, we found that the loss of METTL3 significantly inhibited the translation of CYP19A1 mRNA, resulting in the irreversible loss of invasion and migration abilities in cells, further highlighting the involvement of METTL3 in the translation regulation of CYP19A1.

After observing the METTL3-mediated translation regulation of CYP19A1, we attempted to elucidate the specific mechanism. Referring to studies of downstream targets regulated by METTL3 in tumours, it has been reported that in gastric cancer, METTL3 promotes ZMYM1 mRNA stability through m^6^A modification, subsequently enhancing its protein expression through the m^6^A reader protein HuR (also known as ELAVL1) [36]. In colorectal cancer, METTL3 methylates SOX2 mRNA, allowing it to bind to the mRNA-binding protein IGF2BP2 at m^6^A sites, preventing SOX2 mRNA degradation and promoting protein expression [37]. To further confirm the regulatory role of METTL3 in the expression of CYP19A1, we conducted MeRIP-qPCR experiments. The findings provided evidence that METTL3 mediates m^6^A modification in CYP19A1 mRNA specifically in the 3’UTR region. However, the involvement of METTL3 and other molecules in the translation of CYP19A1 mRNA in NSCLC remains unclear. To explore the proteins bound to METTL3 during its participation in the regulation of CYP19A1 translation, we conducted immunoprecipitation experiments to enrich proteins interacting with METTL3 using METTL3 antibodies and then identified the types of proteins using proteomic analysis. Through GO pathway enrichment, we found that METTL3 can interact with molecules in the translation initiation complex, including eIF4E, eIF3e, and eIF3i. These translation initiation complex components are crucial for the regulation of translation. eIF4E, as a cap-binding subunit of the eIF4F complex, is essential for cap-dependent translation of all nuclear-encoded mRNAs [38, 39]. eIF3e, as a subunit of the eIF3 complex, promotes the interaction between MNKs and eIF4G, leading to phosphorylation of eIF4E by MNKs and subsequently exerting translational regulation functions [40]. Recent studies have also shown increased expression of eIF3i, another subunit of the eIF3 complex, in various tumours, indicating its involvement in abnormal translation processes [41, 42]. Therefore, the binding of METTL3 to translation initiation factors suggests its close association with the mRNA translation process. Furthermore, to explore the direct association between METTL3 and CYP19A1 mRNA, based on the mechanism of METTL3-mediated translation regulation through mRNA circularization, we conducted RNA immunoprecipitation experiments and found that METTL3 directly interacts with CYP19A1 mRNA. Additionally, to confirm the direct involvement of eIF4E in the translation process of CYP19A1, we used the eIF4E/eIF4G interaction inhibitor 4EGI-1 to block eIF4E-mediated translation initiation and observed a significant decrease in CYP19A1 protein levels. These experiments support the mechanism by which METTL3 enhances translation by binding to translation initiation factors and CYP19A1 mRNA, thereby facilitating ribosome cycling and enhancing translation [43].

However, this study has some limitations. For example, we did not extensively investigate the phenomenon of increased CYP19A1 mRNA levels after knocking down or knocking out METTL3 in this study. However, relevant literature has reported similar findings: the knockdown of METTL3 leads to a decrease in m^6^A levels in Snail mRNA, resulting in reduced mRNA degradation mediated by the reader protein YTHDF2 and an increase in Snail mRNA levels. Therefore, the increase in CYP19A1 mRNA observed in this study may be due to a similar mechanism. Additionally, although the effects of STM2457 on proliferation and metastasis of lung cancer cells have been demonstrated already [44, 45], further exploration is warranted regarding its effects on immune phenotypes. Whether this drug can exert synergetic therapeutic effects on NSCLC with immunotherapy and its potential for clinical translation requires in-depth research. It is also worth exploring the molecular expression profiles and downstream signalling pathway changes induced by STM2457 intervention in NSCLC using high-throughput sequencing methods.

In conclusion, through translatomics data mining and experiments, we verified that METTL3 activates the oestrogen signalling pathway to mediate the invasion and metastasis of NSCLC by enhancing the translation of CYP19A1. The specific mechanism is that enzyme activity-dependent METTL3 interacts with translation initiation factors and binds CYP19A1 mRNA, thus enhancing the translation efficiency of CYP19A1. Therefore, this study clarified a new mechanism of metastasis promoted by METTL3, provided a new target for inhibiting NSCLC metastasis, and introduced a new strategy for treating NSCLC metastasis.

### Electronic supplementary material

Below is the link to the electronic supplementary material.

Supplementary Material 1 (Table S1–S4): Gene expression data correlated with clinical information, sequencing data, and mass spectometry data. Table S1: METTL3/CYP19A1 expression of TMA data from 84 NSCLC patients; Table S2: METTL3 expression in DIA-MS data from a late-stage NSCLC cohort (PMID 34870237); Table S3: Genes in each group of the venn diagram in Figure 4C; Table S4: List of mass spectrometry data of each group in Figure 7A

Supplementary Material 2: (Fig. S1–S4): Phenotyes of METTL3 knockdown, expression and prognostic value of ERβ, and RIP-qPCR comfirmation results. Fig. S1: METTL3 promotes migration, invasion and invadopodia formation in NSCLC cells; Fig. S2: Expression and prognostic value of ERβ in NSCLC; Fig. S3: DNA agarose gel electrophoresis analysis in H1975 cell lines to confirm the RIP-qPCR results

## Data Availability

The Polysome profiling data used in this study have been deposited in the Gene Expression Omnibus (GEO) under accession number GSE117299. All other relevant data are available from the corresponding author on request.
