# Supplementary material for: METTL3 drives NSCLC metastasis by enhancing CYP19A1 translation and oestrogen synthesis
Source: Cell Biosci. 2024 Jan 18;14:10. doi: 10.1186/s13578-024-01194-9 (PMC10795463; doi:10.1186/s13578-024-01194-9)
Supplement: Supplementary file 2 — Supplementary Material 2: (Fig. S1–S4): Phenotyes of METTL3 knockdown, expression and prognostic value of ERβ, and RIP-qPCR comfirmation results. Fig. S1: METTL3 promotes migration, invasion and invadopodia formation in NSCLC cells; Fig. S2: Expression and prognostic value of ERβ in NSCLC; Fig. S3: DNA agarose gel electrophoresis analysis in H1975 cell lines to confirm the RIP-qPCR results [file 13578_2024_1194_MOESM2_ESM.docx]

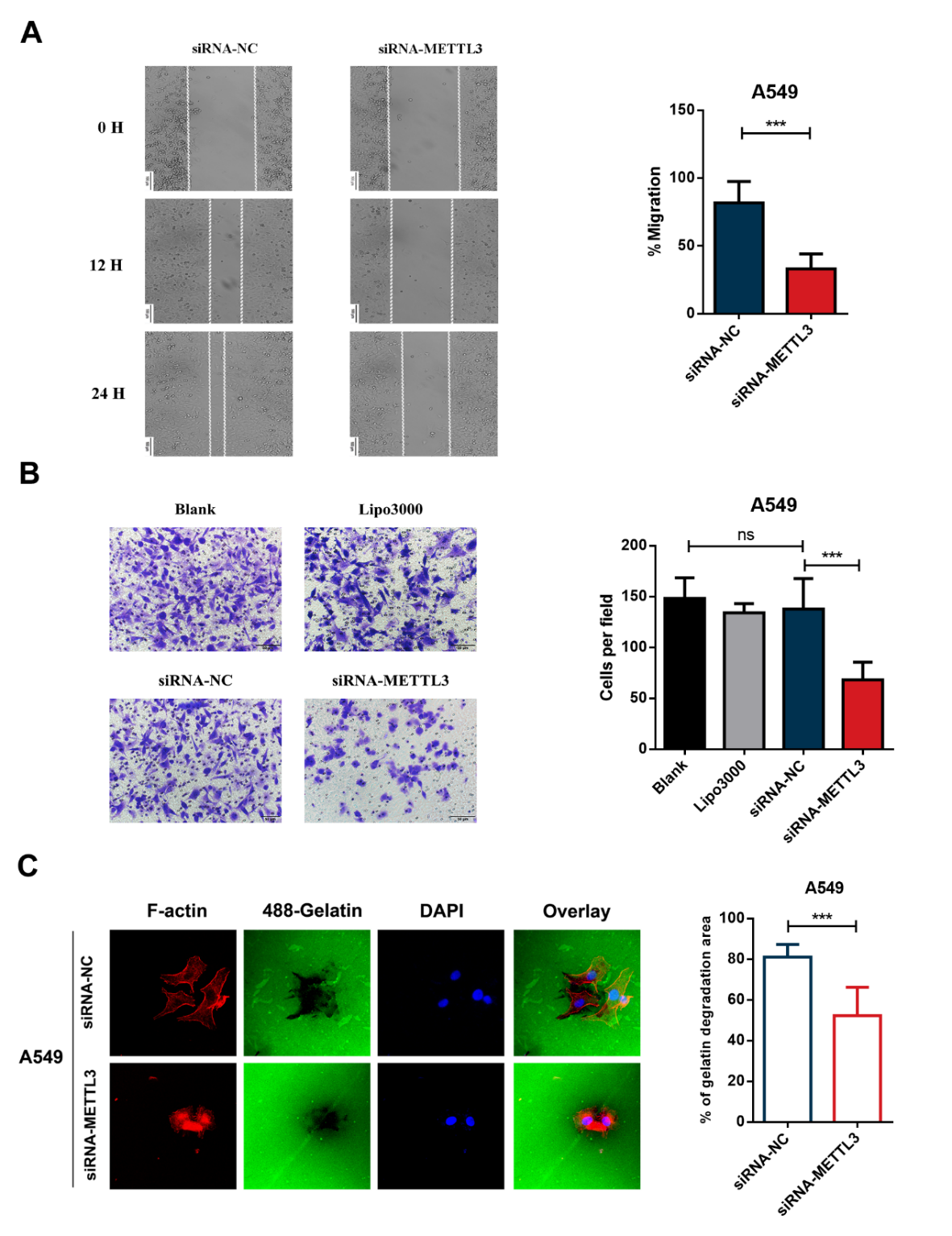


**Figure S1. METTL3 promotes migration, invasion and invadopodia formation in NSCLC cells.** A. After transfection with siRNA targeting METTL3, the wound healing experiment showed a significant decrease in the migration ability of A549 cells. B. Transwell invasion experiments following transfection with siRNA targeting METTL3 showed a significant decrease in invasion ability of A549 cells. C. In the fluorescence matrix degradation experiment, the depth and extent of extracellular matrix degradation were reduced in A549 cells after transfection with siRNA targeting METTL3.


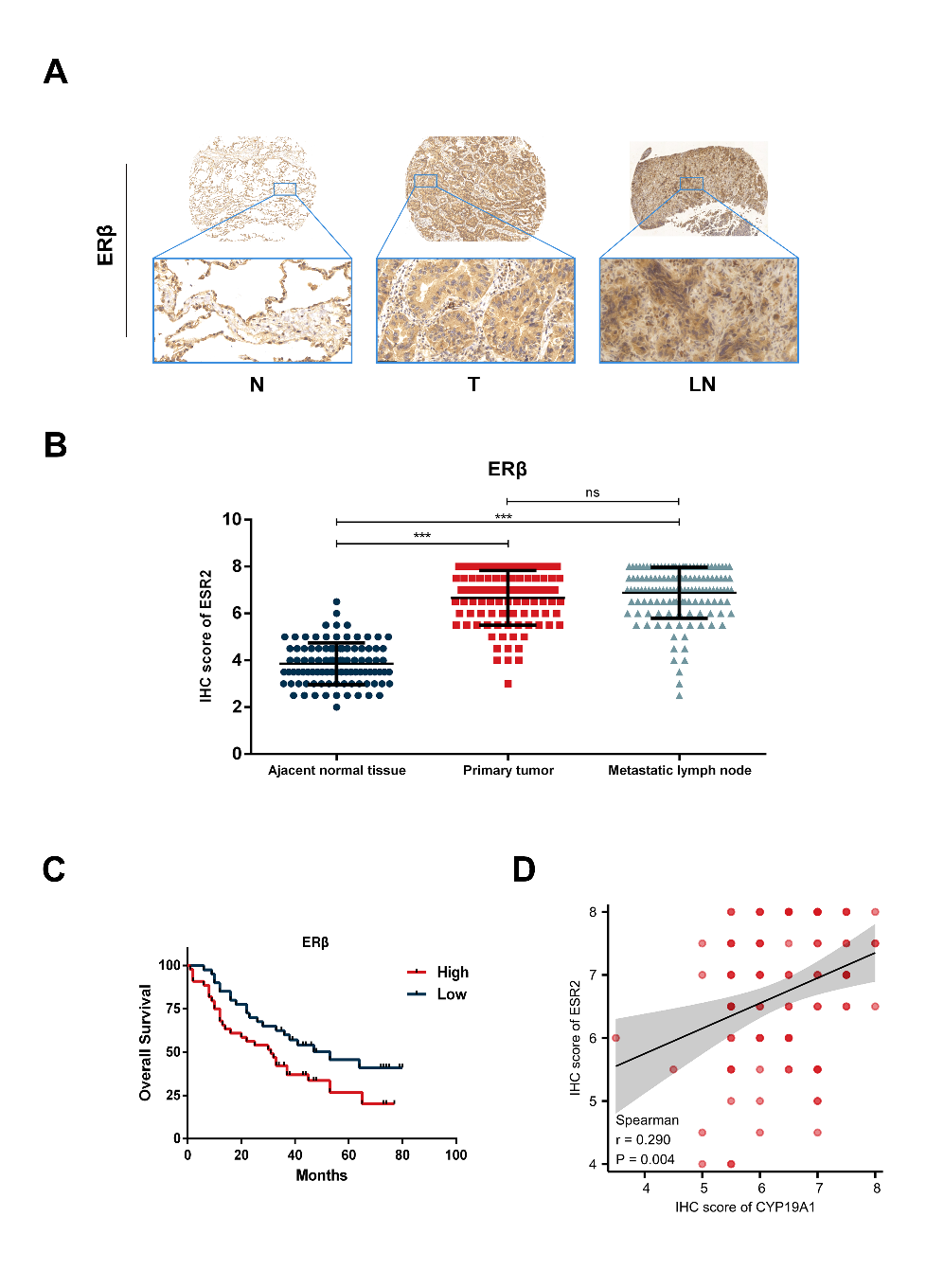


**Figure S2.** **Expression and prognostic value of ERβ in NSCLC.** A. IHC staining of a tissue microarray from 84 patients indicates high expression of ERβ in primary lesions and metastatic lymph nodes of NSCLC. B. Quantification results of IHC staining in tissue microarrays. C. Survival analysis demonstrates the relationship between ERβ expression in tissue microarrays and the prognosis of NSCLC patients, with high expression of ERβ suggesting poor prognosis. D. The results of IHC analysis indicated that CYP19A1 expression was positively correlated with ERβ expression in NSCLC.


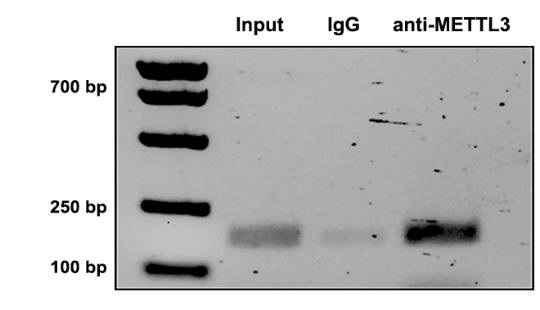


**Figure S3: DNA agarose gel electrophoresis analysis in H1975 cell lines to confirm the RIP-qPCR results**
